# Electron Microscopic Radioautographic Study on the Protein Synthesis in the Pancreas of Aging Mice With Special Reference to Mitochondria

**DOI:** 10.4021/gr310e

**Published:** 2011-05-20

**Authors:** Tetsuji Nagata

**Affiliations:** Department of Anatomy and Cell Biology, Shinshu University School of Medicine, Matsumoto 390-8621, Matsumoto, and Department of Anatomy, Shinshu Institute of Alternative Medicine and Welfare, Nagano 380-0816, Japan. Email: nagata@kowagakuen.ac.jp

**Keywords:** Electron microscopy, Radioautographology, Mitochondrion, Protein synthesis, Pancreas, Acinar cell

## Abstract

**Background:**

The purpose of this study was to investigate the aging changes of macromolecular synthesis in animal cells.

**Methods:**

We studied 10 groups of mice during development aged from fetal day 19 to postnatal month 24. They were injected with ^3^H-leucine, a precursor for protein synthesis, sacrificed and the pancreatic tissues were taken out, fixed and processed for light and electron microscopic radioautography. On many radioautograms the localization of silver grains demonstrating protein synthesis in pancreatic acinar cells in respective aging groups were first analyzed qualitatively. Then the number of silver grains and the number of cell organelles in each cell in respective aging groups were analyzed quantitatively in relation to the aging of animals. The number of mitochondria, the number of labeled mitochondria and the mitochondrial labeling index labeled with silver grains were counted in each pancreatic acinar cell.

**Results and Conclusions:**

The number of silver grains in cell nuclei and cell organelles changed with the aging of animals. The number of mitochondria, the number of labeled mitochondria and the labeling indices showing protein synthesis at various ages increased from embryonic day 19 to postnatal newborn day 1, 3, 7, 14, to young adult month 1, and 2, reaching the maxima, then decreased at old adult month 6 and senile year 1 to 2, indicating the aging changes.

## Introduction

The pancreas is a large gland, next to the liver, among the digestive glands connected to the intestines in the bodies of experimental animals mice and rats. It consists of exocrine and endocrine portions and takes the shape of a compound acinous gland. The exocrine portion is composed of ductal epithelial cells, centro-acinar cells, acinar cells and connective tissue cells, while the endocrine portion, the islet of Langerhans, is composed of 3 types of endocrine cells, A, B, C cells and connective tissue cells [1].

In addition to the DNA and RNA syntheses in nuclei and nucleoli in various cells of aging mice, we also found the silver grains due to DNA and RNA synthesis in mitochondria of various isolated cells such as the liver and kidney cells in vitro showing intramitochondrial DNA and RNA syntheses [2-4]. We later found that the activities of DNA and RNA syntheses in mitochondria of various cells changed due to aging of individual animals [5-7].

Thus, we have recently concentrated to clarify the intramitochondiral DNA and RNA as well as protein syntheses in various cells of aging mice [8], especially in the liver which contains many mitochondria [9]. This paper deals with the intramitochondrial protein synthesis in pancreatic acinar cells of aging ddY mice at various ages in 10 groups during development, aging from prenatal embryos to postnatal 2 years at senescence.

## Materials and Methods

### Experimental animals

The pancreatic tissues were obtained from 10 groups of aging normal ddY strain mice, each consisting of 3 litter mates of both sexes, total 30, from prenatal embryo day 19 to newborn postnatal day 1, 3, 7, 14, adult at month 1, 2, 6, 12 (year 1) to month 24 (year 2). All the animals were housed under conventional conditions and bred with normal diet (mouse chow Clea EC2, Clea Co., Tokyo, Japan) with access to water ad libitum in our laboratory. They were administered with ^3^H-leucine, a protein precursor, and the pancreatic tissues were taken out, fixed and processed for electron microscopic radioautography. All the procedures used in this study concerning the animal experiments were in accordance with the guidelines of the animal research committee of Shinshu University School of Medicine as well as the principles of laboratory animal care in NIH publication No. 86-23 (revised 1985).

### Procedures of electron microscopic radioautography

All the animals were injected intraperitoneally with ^3^H-leucine (Amersham, England, specific activity 877 GBq/mM) in saline, at 9 a.m., one hour before sacrifices. The dosage of injections was 370 KBq/gm body weight. The animals were perfused at 10 a.m., one hour after the injection, via the left ventricles of the hearts with 0.1 M cacodylate-buffered 2.5% glutaraldehyde under Nembutal (Abbott Laboratories, Chicago, ILL, USA) anesthesia. The right end of the pancreatic gland was taken out from each animal, excised and 3 small pieces of the pancreatic tissues (size 1mm × 1mm × 1mm) were immersed in the same fixative at 4 °C for 1 hr, followed by postfixation in 1% osmium tetroxide in the same buffer at 4 °C for 1 hr, dehydrated in graded series of ethanol and acetone, and embedded in epoxy resin Epok 812 (Oken, Tokyo, Japan).

For electron microscopic radioautography, semithin sections at 0.2 µm thickness, thicker than conventional ultrathin sections in order to shorten the exposure time, were cut in sequence on a Porter-Blum MT-2B ultramicrotome (Dupont-Sorvall, Newtown, MA, USA) using glass knives. The sections were collected on collodion coated copper grid meshes (VECO, Eerbeek, Netherlands), coated with Konica NR-H2 radioautographic emulsion (Konica, Tokyo, Japan) by a wire-loop method [10]. They were stored in dark boxes containing silica gel (desiccant) at 4 °C for exposure. After the exposure for 10 months, the specimens were processed for development in freshly prepared gold latensification solution for 30 sec at 16 °C and then in fresh phenidon developer for 1 min at 16 °C in a water bath, rinsed in distilled water and dried in an oven at 37 °C overnight, stained with lead citrate solution for 3 min, coated with carbon for electron microscopy. The electron microscopic (EM) radioautograms were examined in a JEOL JEM-4000EX electron microscope (JEOL, Tokyo, Japan) at accelerating voltages of 400 kV for observing thick specimens [8, 9].

### Quantitative analysis of electron micrographs

For quantitative analysis of electron micrographs, twenty EM radioautograms showing cross sections of pancreatic acinar cells from each group, based on the electron microscopic photographs taken after observation on at least 100 pancreatic acinar cells from respective animals were analyzed to calculate the total number of mitochondria in each cell, and the number of labeled mitochondria covered with silver grains by visual grain counting.

On the other hand, the number of silver grains in the same area size as a mitochondrion outside cells was also calculated in respective specimens as background fog, which resulted in less than 1 silver grain (0.02/mitochondrial area) almost zero. Therefore, the grain count in each specimen was not corrected with background fog. From all the data thus obtained the averages and standard deviations in respective aging groups were computed with a personal computer (Macintosh type 8100/100, Apple Computer, Tokyo, Japan). The data were stochastically analyzed using variance and Student’s t-test. The differences were considered to be significant at P value < 0.01.

## Results

### Morphological observations

**Figure 1:**
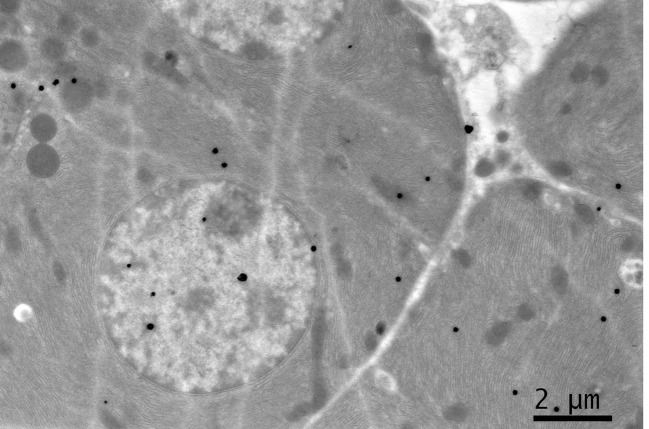
EM RAG of 2 pancreatic acinar cells of a newborn day 1 mouse labeled with ^3^H-leucine, showing a few silver grains in the nucleus and cytoplasm (× 3,000).

**Figure 2:**
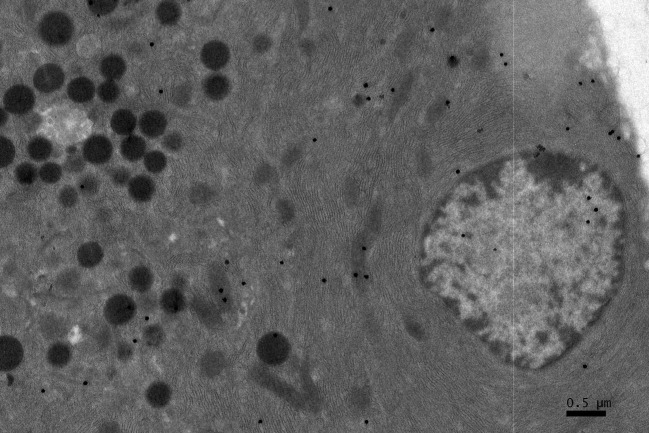
EM RAG of a pancreatic acinar cell of a postnatal day 7 mouse labeled with ^3^H-leucine (× 3,000).

**Figure 3:**
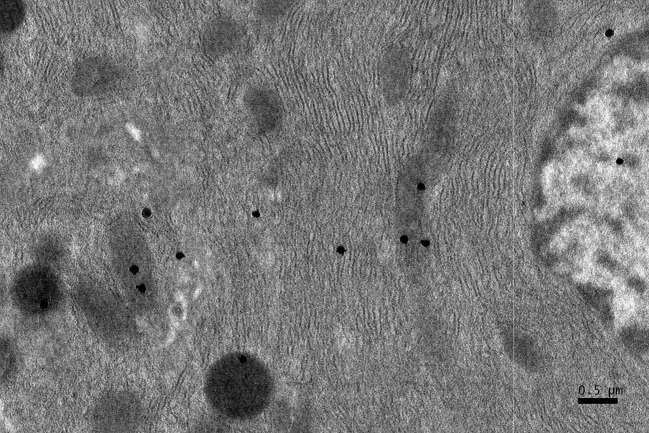
A high power magnification EM RAG of a pancreatic acinar cell of a postnatal day 14 mouse containing a nucleus and several mitochondria labeled with ^3^H-leucine, showing several silver grains in a few mitochondria (× 6,000).

**Figure 4:**
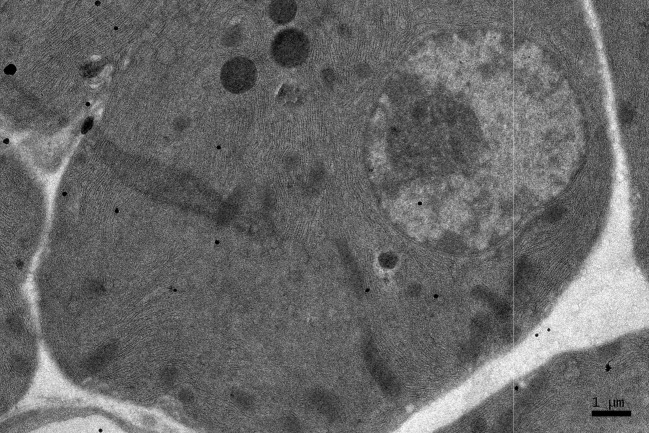
EM RAG of a pancreatic acinar cell of a postnatal month 6 mouse labeled with ^3^H-leucine, showing several silver grains on several mitochondria and cytoplasmic matrix (× 6,000).

**Figure 5:**
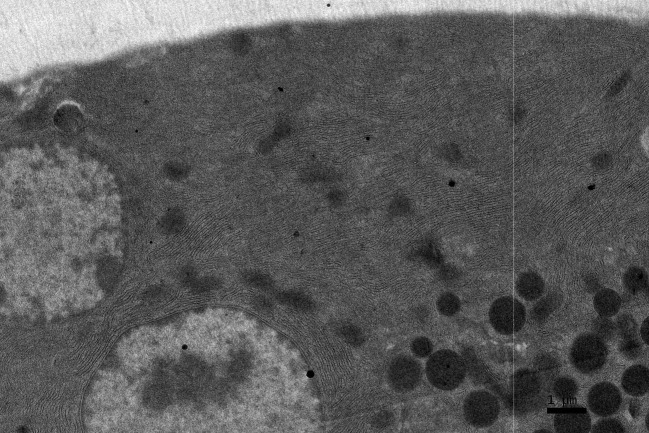
EM RAG of a pancreatic acinar cell of a postnatal month 24 mouse labeled with ^3^H-leucine, showing several silver grains over one of the 2 nuclei as well as over a few mitochondria and endoplasmic reticulum (× 6,000).

**Figure 6:**
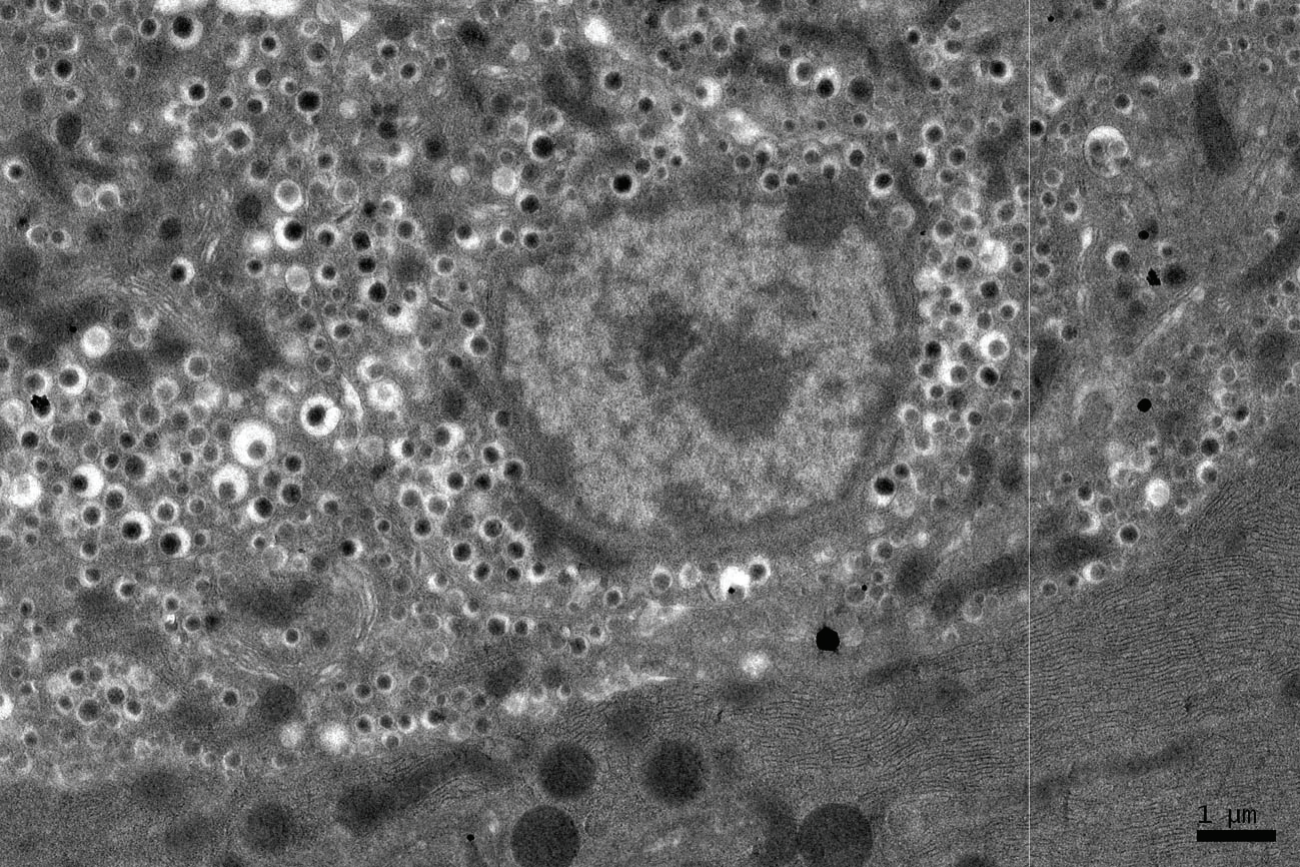
EM RAG of an endocrine cell (the islet of Langerhans) and a pancreatic acinar cell of a postnatal month 24 mouse labeled with ^3^H-leucine, showing less silver grains in the endocrine cell than the exocrine cell (× 3,000).

The pancreatic tissues obtained from ddY strain mice at various ages from embryo day 19 to postnatal month 24, consisted of 2 portions, the exocrine portion (Fig. 1-5) and the endocrine portion or designated as the islets of Langerhans (Fig. 6). The exocrine portion is consisted of several cell types, the pancreatic acinar cells (Fig. 1-5), the centroacinar cells, ductal cells and fibroblasts, as observed by electron microscopy. The acinar cells are main components of the exocrine portions which contain well developed endoplasmic reticulum, zymogen granules, and many mitochondria in the cytoplasm. Because the number of mitochondria in the pancreatic acinar cells was relatively much more than the other cells, only the pancreatic acinar cells were analyzed in this study.

### Radioautographic observations

Observing electron microscopic radioautograms, the silver grains were found over the nuclei as well as over the cytoplasm including mitochondria of some pancreatic acinar cells (Fig. 1-5), labeled with ^3^H-leucine, demonstrating protein synthesis at respective aging stages from perinatal stage at embryonic day 19 (Fig. 1), to postnatal day 1 and day 3 and 7 (Fig. 2), and day 14 (Fig. 3), to adult stage at month 1, month 2 and 6 (Fig. 4), and to senescent stage at month 12 and 24 (Fig. 5).

The localizations of silver grains over the mitochondria were mainly on the mitochondrial matrices similarly to other cells such as in the livers [9] or the adrenal glands [11].

### Quantitative analysis

#### Number of mitochondria per cell

**Figure 7:**
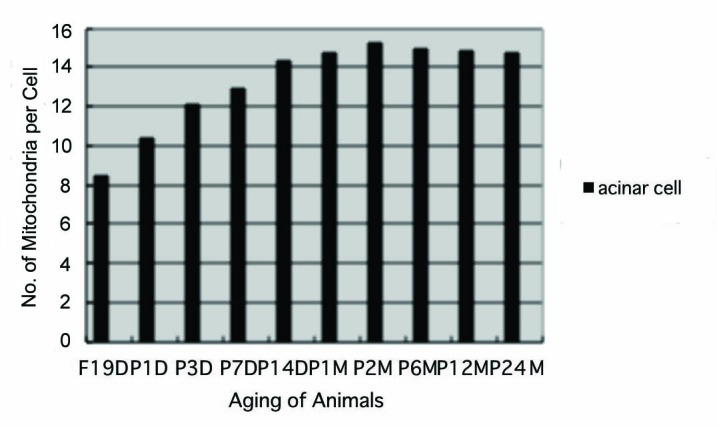
Histogram showing the number of mitochondria per cell in respective aging groups labeled with ^3^H-leucine.

Preliminary quantitative analysis on the number of mitochondria in 10 pancreatic acinar cells whose nuclei were labeled with silver grains and other 10 cells whose nuclei were not labeled in each aging group revealed that there was no significant difference between the number of mitochondria and the labeling indices (P < 0.01). Thus, the number of mitochondria and the labeling indices were calculated regardless whether their nuclei were labeled or not. The results obtained from the number of mitochondria in pancreatic acinar cells of respective animals in 10 aging groups at perinatal and newborn stages, from prenatal embryo day 19 to postnatal day 1, 3, 7, 14, and adult and senescent stages at month 1, 2, 6, 12, and 24, seemed to show a gradual increase from the prenatal day 19 to postnatal month 24. The numbers of zymogen granules increased due to the aging from prenatal embryo to postnatal day 1, 3, 7, 14 and to month 1, 2, reaching the maximum and did not increase to month 6, 12 and 24. The number of mitochondria per cell at respective aging stages, on the other hand, increased from prenatal embryo around 8.5/cell in average, to 10.4 at postnatal day 1, to 12.1 at day 3, to 12.9 at day 7, to 14.3 at day 14, to 14.7 at month 1, to 15.2 at month 2, then slightly decreased to 14.9 at month 6, to 14.8 at month 12 and finally to 14.7 at month 24 as shown in Figure 7. All the data from embryonic day 19 to postnatal month 24, were stochastically analyzed using variance and Student’s t-test. The increase of mitochondrial numbers in the pancreatic acinar cells from embryonic day 19 to adult stage at postnatal month 2 was considered to be significant at P value < 0.01. However, the slight decrease at the senescent stage from month 6 to 24 was considered to be not significant at P value < 0.01.

#### Mitochondrial protein synthesis

**Figure 8:**
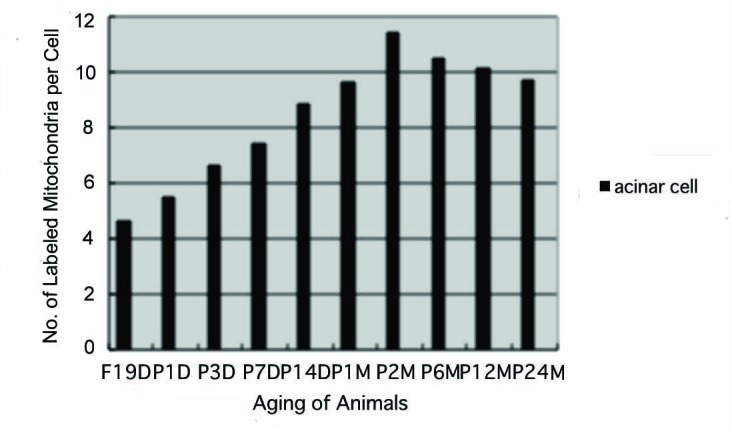
Histogram showing the number of labeled mitochondria per cell in respective aging groups labeled with ^3^H-leucine.

The results of visual counting on the number of mitochondria labeled with silver grains obtained from 10 pancreatic acinar cells of each animal labeled with ^3^H-leucine demonstrated protein synthesis in 10 aging groups, from prenatal embryo day 19 (4.6/cell), increased gradually to postnatal day 1 (5.5), day 3 (6.6) day 7 (7.4) and day 14 (8.8), to month 1 (9.6), month 2 (11.4), reaching the maximum, then decreased gradually to month 6 (10.5), month 12 (10.1) and month 24 (9.7/cell) as shown in Figure 8. The data were stochastically analyzed using variance and Student’s t-test. The increase of the numbers of labeled mitochondria from embryo day 19 to postnatal month 2 was stochastically significant (P < 0.01). However, the decrease from month 2 to month 24 was not significant.

#### The labeling index

**Figure 9:**
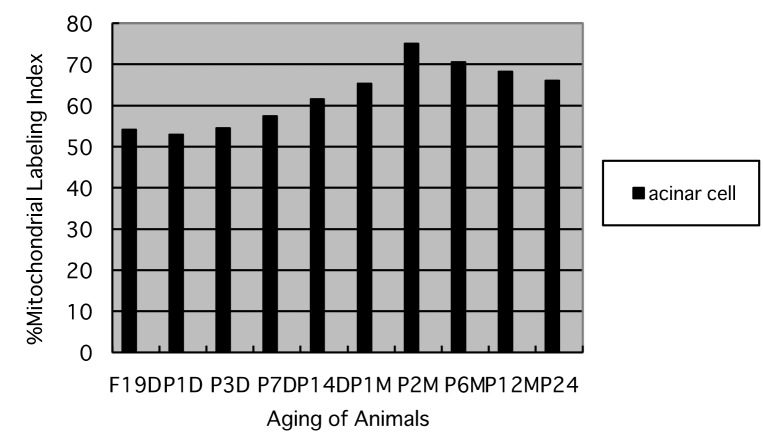
Histogram showing the average labeling indices in respective aging groups labeled with ^3^H-leucine.

Finally, the labeling indices of mitochondrial protein synthesis in pancreatic acinar cells at respective aging stages were calculated through dividing the number of labeled mitochondria (Fig. 8) by the number of total mitochondria per cell (Fig. 7) which were plotted in Figure 9.

The results showed that the labeling indices gradually increased from prenatal day 19 (54.1%) and postnatal newborn day 1 (52.9%), to postnatal day 3 (54.5%), day 7 (57.4%), day 14 (61.5%), to adult stages at month 1 (65.3%), month 2 (75.1%), month 6 (70.5%), reaching the maximum, and then decreased at month 12 (68.2%) and 24 (66.1%) as shown in Figure 9. From the results, the increases of the mitochondrial labeling indices in pancreatic cells from embryo day 19 and newborn postnatal day 1 to postnatal day 7 to month 6, as well as the decreases from month 6 to month 12 and 24 were stochastically significant (P < 0.01).

## Discussion

We have studied the macromolecular synthesis of the aging mouse pancreas at various ages since many years. We first studied the DNA synthesis of mouse pancreas by LM and EMRAG using ^3^H-thymidine [1]. Light and electron microscopic radioautograms of the pancreas revealed that the nuclei of pancreatic acinar cells, centro-acinar cells, ductal epithelial cells, and endocrine cells were labeled with ^3^H-thymidine. The labeling indices of these cells in 5 groups of litter mate mice, fetal day 15, postnatal day 1, 20, 60 and 700 (2 years) were analyzed. The labeling indices of these cells reached the maxima at day 1 after birth and decreased gradually to 2 years. The maximum in the acinar cells proceeded to the ductal and centro-acinar cells, suggesting that the acinar cells completed their development earlier than the ductal and centro-acinar cells [1, 12].

On the other hand, LM and EMRAG of pancreas of mouse injected with ^3^H-uridine demonstrated its incorporation into exocrine and then in endocrine cells, and more in pancreatic acinar cells than in ductal or centro-acinar cells. Among the acinar cells, the number of silver grains increased after birth to day 14 and then decreased with aging [12]. Quantification of silver grains in the nucleoli, chromatin, and cell body were carried out by X-ray microanalysis [12], which verified the results obtained by visual grain counting. In EMRAG obtained from the pancreas of fetal day 19 embryos, newborn day 1 and newborn day 14 mice labeled with ^3^H-uridine, demonstrating RNA synthesis, the number of silver grains in the nucleoli, nuclear chromatin and cytoplasm increased. In order to quantify the silver contents of grains observed over the nucleoli, nuclei and cytoplasm, X-ray spectra were recorded by energy dispersive X-ray microanalysis (JEM-4000EX TN5400), demonstrating Ag-Kα peaks at higher energies. Thus, P/B ratios expressing relative silver contents were determined and compared between the two age groups. On the other hand, the results obtained by visual grain counting in different cell compartments in day 1 and day 14 animals were also listed. The number of silver grains was calculated to express the counts per unit area to be compared with the XMA counts. These two results, the silver content analyzed by X-ray microanalysis and the results obtaining from visual grain counting were in good accordance with each other.

As for the protein synthesis in the pancreas, ^3^H-leucine incorporation into endoplasmic reticulum, Golgi apparatus and to secretory granules of pancreatic acinar cells was first demonstrated by Jamieson and Palade [13]. We first studied ^3^H-glycine incorporation into these cell organelles of mouse pancreatic acinar cells in connection with soluble compounds by EMRAG [14]. It was demonstrated that soluble ^3^H-glycine distributed not only in these cell organelles but also in the karyoplasm and cytoplasm diffusely. Then, the quantitative aspects of protein synthesis with regard to the aging from fetal day 19, to postnatal day 1, 3, 7, 14 and 1, 2, 5 and 12 months were also clarified. The results showed an increase of silver grain counts labeled with ^3^H-leucine after birth, reaching a peak from postnatal 2 weeks to 1 month, and decreasing from 2 months to 1 year.

Concerning the glucide synthesis, we first studied the incorporation of ^3^H-glucose into the pancreatic acinar cells of mouse in connection with soluble compounds by EMRAG [14]. It was demonstrated that soluble ^3^H-glucose distributed not only in such cell organelles as endoplasmic reticulum, Golgi apparatus, mitochondria but also in the karyoplasm and cytoplasm diffusely. Then, the incorporation of ^3^H-glucosamine into the pancreas of aging mice at various ages was studied by LM and EMRAG [10]. When perinatal baby mice received ^3^H-glucosamine injection and the pancreatic tissues were radioautographed, silver grains were observed over exocrine and endocrine pancreatic cells. However, the number of silver grains was not so many. When juvenile mice at the age of 14 days after birth were examined, many silver grains appeared over the exocrine pancreatic acinar cells. Less silver grains were observed over endocrine pancreatic cells and ductal epithelial cells. The grains in the exocrine pancreatic acinar cells were localized over the nucleus, endoplasmic reticulum, Golgi apparatus and secretory granules, demonstrating glycoprotein synthesis. Adult mice at the ages of 1 month, 6 month old or senile mice at the ages of 12 months or 24 months showed very few silver grains on radioautograms. Thus, the glucide synthesis in the pancreas of mice revealed quantitative changes, an increase and a decrease of ^3^H-glucosamine incorporation with aging [10].

In order to demonstrate lipid synthesis, several litters of ddY mice aged fetal day 19, postnatal day 1, 3, 7, 14, and 1, 2, 6 up to 12 months, were injected with ^3^H-glycerol and the pancreases were prepared for LM and EMRAG. The silver grains were observed in both exocrine and endocrine cells of respective ages. In perinatal animals from fetal day 19 to postnatal 1, 3, and 7 days, cell organelles were not well developed in exocrine and endocrine cells and number of silver grains was very few. In 14 day old juvenile animals, cell organelles such as endoplasmic reticulum, Golgi apparatus, mitochondria and secretory granules were well developed and many silver grains were observed over these organelles and nuclei in both exocrine and endocrine cells. The number of silver grains was more in exocrine cells than in endocrine cells. In 1, 2, 6 month old adult animals, number of silver grains remained constant. In 12 month old senescent animals, silver grains were fewer than younger animals. The number of silver grains expressed the quantity of lipids synthesis, which increased from perinatal period to adult and decreased to senescence.

On the other hand, experimental studies to clarify the pathogenesis of experimental pancreatitis were carried out comparing with the normal mouse pancreas by means of LM and EMRAG. Several male Wistar rats were treated with daily DL-ethionine injections for 2 days or for 1 month to cause the acute and chronic experimental ethionine pancreatitis. Then, the pancreatic tissues from both normal and experimental pancreatitis animals were taken out and incubated in vitro in a medium containing ^3^H-ethionine and the tissues were fixed after 5, 10, 30, 50 and 120 min culture and processed for LM and EMRAG [15]. As the results, less silver grains were observed over the secretory granules and the lumen of the exocrine pancreatic tissues of normal control animals than the experimental pancreatitis animals, suggesting the slow intracellular transport of ethionine in normal animals than the ethionine induced pancreatitis animals [15].

Another group of several male Wistar rats were fed with 20% ethanol for 3 months to cause alcoholic pancreatitis. Both the normal and experimental pancreatitis animals were then injected with ^3^H-leucine and the pancreatic tissues after 5 to 60 minutes of injection were fixed and processed for LM and EMRAG [16]. As the results, more silver grains were observed over the secretory granules and the lumen of the exocrine pancreatic tissues of normal animals than the pancreatitis, suggesting the higher protein synthetic activity in normal animals than the alcohol induced pancreatitis animals [16].

On the other hand, our previous papers were the first which deal with the relationship between the DNA synthesis and aging in hepatocytes of mice in vivo at various ages by means of electron microscopic radioautography observing the small dot-like silver grains, due to incorporations of ^3^H-thymidine, which exactly localized inside the mitochondria [9, 17, 18]. Later we also studied intramitochondrial DNA synthesis in adreno-cortical cells from prenatal day 19 to postnatal day 1, 3, 9, 14, month 1, 2, 6, 12 and 24 (year 2) and found that the numbers of mitochondria in 3 zones, glomerulosa, fasciculate and reticularis, increased reaching the maxima at postnatal month 2 which kept continued until senescence up to 24 months (2 years). On the contrary, the numbers of labeled mitochondria and the labeling indices increased to postnatal month 2, reaching the maxima, then decreased to month 24 [19, 20].

Later we also demonstrated the results from the RNA synthesis in the livers and adrenal glands of aging mice which also revealed that an increase was observed by direct observation on mitochondria at electron microscopic level and obtained accurate mitochondrial number and labeling indices in the hepatocytes and adreno-cortical and adreno-medullary cells. In the present study, we also demonstrated the protein synthesis in the pancreatic acinar cells in 10 groups of developing and aging mice. There was a discrepancy between our results from the hepatocytes [21-26], the adrenal cells [19, 20] as well as the pancreatic acinar cells at present [9, 11] and the results from the several types of cells in the brains by Korr and associates [27-30]. The reason for this difference might be due to the difference between the cell types (hepatocytes, adrenal cells, pancreatic cells from our results and the brain cells from their results) or the difference between the observation by light or electron microscopy, i.e., direct observation of mitochondrial structure by electron microscopy in our results or light microscopy, or indirect observation of mitochondria without observing any mitochondrial structure directly by Korr et al. [27, 28].

Anyway, the results obtained from the pancreartic acinar cells of aging mice at present should form a part of special cytochemistry [31] in cell biology, as well as a part of special radioautographology [8], i.e., the application of radioautography to the pancreas, as was recently reviewed by the present author including recent results dealing with various organs. We expect that such special radioautographology and special cytochemistry should be further developed in all the organs in the future.

### Conclusion

From the results obtained at present, it was concluded that almost all the pancreatic acinar cells in the pancreatic exocrine portions of mice at various ages, from prenatal embryo day 19 to postnatal newborn, day 1, 3, 7 and 14, and to postnatal month 1, 2, 6, 12 and 24, were labeled with silver grains showing protein synthesis with ^3^H-leucine in their mitochondria. Quantitative analysis of the number of mitochondria in pancreatic acinar cells revealed increases from the prenatal day to postnatal day 1, 3, 9, 14, to month 1 and 2, reaching the maximum at postnatal month 2, then slightly decreased to month 6, 12 and 24. Likewise, the numbers of labeled mitochondria with ^3^H-leucine showing protein synthesis and the labeling indices also increased from prenatal day 19 to postnatal day 14, to month 1 and 2, reaching the maximum at postnatal month 2 and decreased to month 6, 12 and 24. These results demonstrated that the number of mitochondria in the pancreatic acinar cells increased from perinatal stages to postnatal month 2, keeping the maximum up to month 24, while the activity of mitochondrial protein synthesis increased to postnatal day 14, to postnatal month 2, reaching the maximum, then decreased to month 24 due to aging of animals.
